# Locational privacy-preserving distance computations with intersecting sets of randomly labeled grid points

**DOI:** 10.1186/s12942-021-00268-y

**Published:** 2021-03-20

**Authors:** Rainer Schnell, Jonas Klingwort, James M. Farrow

**Affiliations:** 1 Research Methodology Group, University of Duisburg-Essen, Duisburg, Germany; 2 Methodology R&D, Statistics Netherlands (CBS), Heerlen, The Netherlands; 3 Farrow Norris, Sydney, Australia

**Keywords:** Geographical data, Geo-referenced data, Geo-masking, Record-linkage, ISGP

## Abstract

**Background:**

We introduce and study a recently proposed method for privacy-preserving distance computations which has received little attention in the scientific literature so far. The method, which is based on intersecting sets of randomly labeled grid points, is henceforth denoted as ISGP allows calculating the approximate distances between masked spatial data. Coordinates are replaced by sets of hash values. The method allows the computation of distances between locations *L* when the locations at different points in time *t* are not known simultaneously. The distance between $L_1$ and $L_2$ could be computed even when $L_2$ does not exist at $t_1$ and $L_1$ has been deleted at $t_2$. An example would be patients from a medical data set and locations of later hospitalizations. ISGP is a new tool for privacy-preserving data handling of geo-referenced data sets in general. Furthermore, this technique can be used to include geographical identifiers as additional information for privacy-preserving record-linkage. To show that the technique can be implemented in most high-level programming languages with a few lines of code, a complete implementation within the statistical programming language R is given. The properties of the method are explored using simulations based on large-scale real-world data of hospitals ($n=850$) and residential locations ($n=13,000$). The method has already been used in a real-world application.

**Results:**

ISGP yields very accurate results. Our simulation study showed that—with appropriately chosen parameters – 99 % accuracy in the approximated distances is achieved.

**Conclusion:**

We discussed a new method for privacy-preserving distance computations in microdata. The method is highly accurate, fast, has low computational burden, and does not require excessive storage.

## Background

The number of statistical microdata sets containing geo-referenced data has increased steadily. For example, at least two US medical surveys (National Ambulatory Medical Care Survey, NAMCS, and the National Hospital Ambulatory Medical Care Survey, NHAMCS) have additional data files containing the distances to the nearest eligible hospital as well as the distances to the nearest eligible hospital with an emergency department [1]. Other CDC (Centers for Disease Control and Prevention) surveys (for example, NHANES, NHCS, NHIS, NIS, NSFG, SLAITS) also contain geocodes. The increasing availability of geographical information has generated a continuous stream of research literature on the effects of geographical disparity on health-related outcomes [2–8].

Generally, surveys with geo-referenced information have restricted data access to guarantee as much respondent privacy as possible. The method introduced here could be used for research applications under privacy legislation such as the General Data Protection Regulation as implemented in different ways among European countries. For example, due to privacy concerns in most countries, survey agencies and official statistics bureaus are often required to separate research data and respondent identifying information [9–11]. Depending on the available spatial resolution, geographical locations could be used to identify a person directly. Therefore, geolocations of survey respondents are usually not included in scientific use files. In many research settings, respondents are assured that directly identifying information (such as names or geolocations) is deleted after data collection. Given this, at least two different scenarios for the use of the suggested technique seem to be plausible:

1. In a cohort study of treatment outcomes, initial healthcare providers’ address is pseudonymised and saved. During the follow-up treatment, the pseudonymised addresses of subsequent health care providers are added to the dataset. Estimated distances of providers can be computed even in those cases, where providers do not exist at the initial data collection time.
2. If no unique person identifiers are available for linking records of the same patient between different organisations, quasi-identifiers such as names and addresses are used for linkage. If these identifiers have to be pseudonymised, computing distances between addresses might help in identifying true links. Therefore, the estimated euclidean distances of addresses between potential links could be used for privacy-preserving record-linkage [11].

An application of the first-mentioned type has already been used in practice [12]. The second type is a natural extension of encoding one-dimensional numerical data for privacy-preserving record-linkage [13]. Since respondents’ spatial mobility in many societies is mostly regional, the additional distance information will increase the precision of linkage procedures.

In this paper, a new method for calculating distances between pseudonymized spatial data is presented, which preserves the original distances between locations (Sect. 2). This method was first presented at a conference by [14], but has not been published previously. In contrast to the presentation, we implement the method, provide the proof of the central equation, simulate effects of parameter choices, and demonstrate a successful application with real-world data.

### Previous approaches

Different approaches for the masking of spatial data have been suggested in the literature. Based on [15], the methods sketched in the review by [16] can be classified into three categories: (1) methods that aggregate spatial points, (2) methods that modify coordinates, and (3) methods that release contextual data only. Examples of the first category include point and areal aggregation. Translation, rotation, scaling, and random perturbation belong to the second group, whereas the release of the distances to the nearest neighbors gives an example of the third category. Two of the latest suggestions can be considered as examples of (2): [17] and [18]. The first approach moves each point into the area of a torus, centered at this point. The second approach uses an embedding of the coordinates. However, here we suggest an entirely different approach.

The work most similar to ours has been published by [19]. Kerschbaum introduced a distance-preserving pseudonymization technique for timestamps and spatial data. For the two-dimensional calculation of the distance between two points, the author generates a regular grid of reference points and assigns a hash value to every grid point. The pseudonymization of a point location *P* is the set of grid points with a certain distance *d* from *P*, together with angle and distance to the point of interest. Using the distance and the angle of the grid points, locations $P_1$ and $P_2$ can be recovered.

In contrast to Kerschbaum’s method, we do not calculate the distance between two points by calculating their distances to one common grid point. Instead, we approximate the distance between two spatial points *P* and *Q* by considering the area of intersection of two circles centered at these points. Furthermore, the angle and the distance are available as plain-text in Kerschbaum’s method, which probably allows the re-identification. Finally, the new method allows the computation of distances between locations when the locations at different points in time are not known simultaneously. For example, the distance between $L_1$ and $L_2$ could be computed even when $L_2$ does not exist at $t_1$ and $L_1$ has been deleted at $t_2$.

## Methods

### Approximation of the distance between two spatial points by intersecting sets of randomly labelled grid points

In this Section, we demonstrate the approximation of the distance between two spatial points in a two-dimensional space, without using information about their exact positions. For this purpose, we approximate the area of intersection between two circles surrounding these points.

**Fig. 1:**
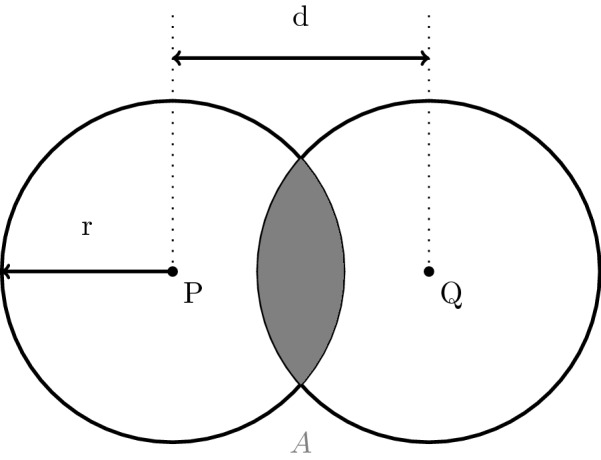
Area of intersection *A* between two circles with the same radius *r*, whose centers *P* and *Q* have distance *d* from each other

Let us consider two points *P* and *Q* and the distance *d* between them. First, we surround each of those points by a circle of radius *r*, as depicted in Fig.  1. Thus, if $0 \le d \le 2r$ holds, the two circles have an area of intersection *A*, which depends on *d*.

Hence, up to a separation of the double radius, there exists a bijective (one-to-one and onto) mapping

$$\begin{aligned} f: [0,2r] \longrightarrow [0,\pi r^{2}], \quad d \mapsto A(d) \end{aligned}$$

between the distance *d* and the area *A* of overlap. It is intuitively clear that every area *A*(*d*) results from exactly one distance $d \in [0,2r]$ between *P* and *Q*. Therefore, we can verify the Equation

1 $$\begin{aligned} A(d) = 2 r^{2} \cdot \arccos (\frac{d}{2r}) - \frac{1}{2} d \cdot \sqrt{4r^{2} -d^{2}} \end{aligned}$$

describing the relation between *A* and *d*. A proof is given in the appendix. Hence, if we know *A* we can approximate *d* as we will show below.

**Fig. 2:**
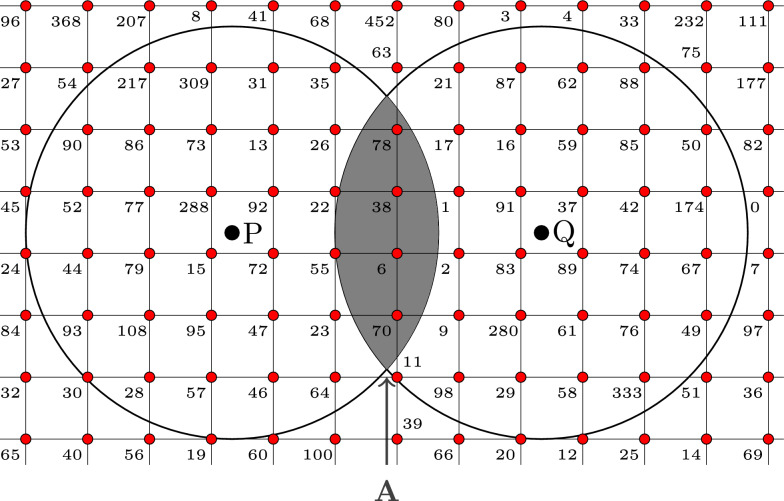
Circles overlaid with a regular grid. Random numbers are assigned to the grid points

Next, we overlay the two circles with a regular grid, as shown in Fig. 2, and map unique random numbers to the grid points. Then, the pseudonymizations ${\mathcal {G}}_{P}$ and ${\mathcal {G}}_{Q}$ of the spatial points *P* and *Q* consist of the grid points surrounded by the respective circle. Furthermore, we determine the set of grid points ${\mathcal {G}}_{P} \cap {\mathcal {G}}_{Q}$ covered by the area of intersection *A*. In the example shown in Fig. 2, this intersection is given by ${\mathcal {G}}_{P} \cap {\mathcal {G}}_{Q} = \{78, 38, 6, 70\}$.

For reasonably flat geometries, like those we consider here, it is sufficient to use a rectangular grid. If the method is extended to curved geometries, like the surface of a sphere, using a triangular grid would provide more accurate results.

Furthermore, the regularity of the grid is important, so that identical distances between considered points yield (dependent on the radius) nearly the same number of grid points enclosed by the area of intersection. In the case of randomly distributed grid points, the accuracy of the result strongly depends on how many grid points are enclosed by the area of intersection. Thus, the error for the approximation of the distance *d* will generally be higher for random grids than for regular grids. We will demonstrate this effect in Sect.  2.

The similarity of the two pseudonymizations for *P* and *Q* can be computed with any suitable similarity measure. Here, we use the Dice coefficient [20], given by

2 $$\begin{aligned} s = \frac{2|{\mathcal {G}}_{P} \cap {\mathcal {G}}_{Q}|}{|{\mathcal {G}}_{P}|+|{\mathcal {G}}_{Q}|}, \end{aligned}$$

where $|\cdot |$ denotes the number of elements contained in the respective set. The similarity measure can then be used to approximate the intersection *A* as proportion of the area $\pi r^{2}$ of a circle through

3 $$\begin{aligned} {\hat{A}} = s \cdot \pi r^2. \end{aligned}$$

Finally, solving the equation $A(d)={\hat{A}}$ yields the approximation for the distance *d* between *P* and *Q*. Since the method is based on intersections of sets of grid points, we denote the procedure as ISGP. We will illustrate ISGP with an application in the next section.

### Step-by-step workflow

In a real-world application as described in the Background (Sect.  1), two data holders could agree on the parameters (seed of pseudo-number random generator, radius, number of grid points, and area). Each of the data holders computes the set of grid points corresponding to the locations of the points of interest (Steps 1–8 in the workflow below). A research group will use these sets of grid points to compute the distances they need for their research (Step 9). The research group only needs the sets of grid points and the information on the radius used for the computation.

We will describe a step-by-step workflow for these steps using the statistical programming language R [21]. As an example, we use two real-world data sets containing geographic information. The first data set contains 850 hospitals located in England.^1^ The second data set is a large administrative database of the United Kingdom containing approximately 13 million residential addresses. As outlined in Sect. 1, the distance to the nearest hospitals is relevant in various research fields. As an example, we will calculate the approximate distances for one residential address to its nearest three hospitals.

#### Step 1: Preprocessing

**Fig. 3:**
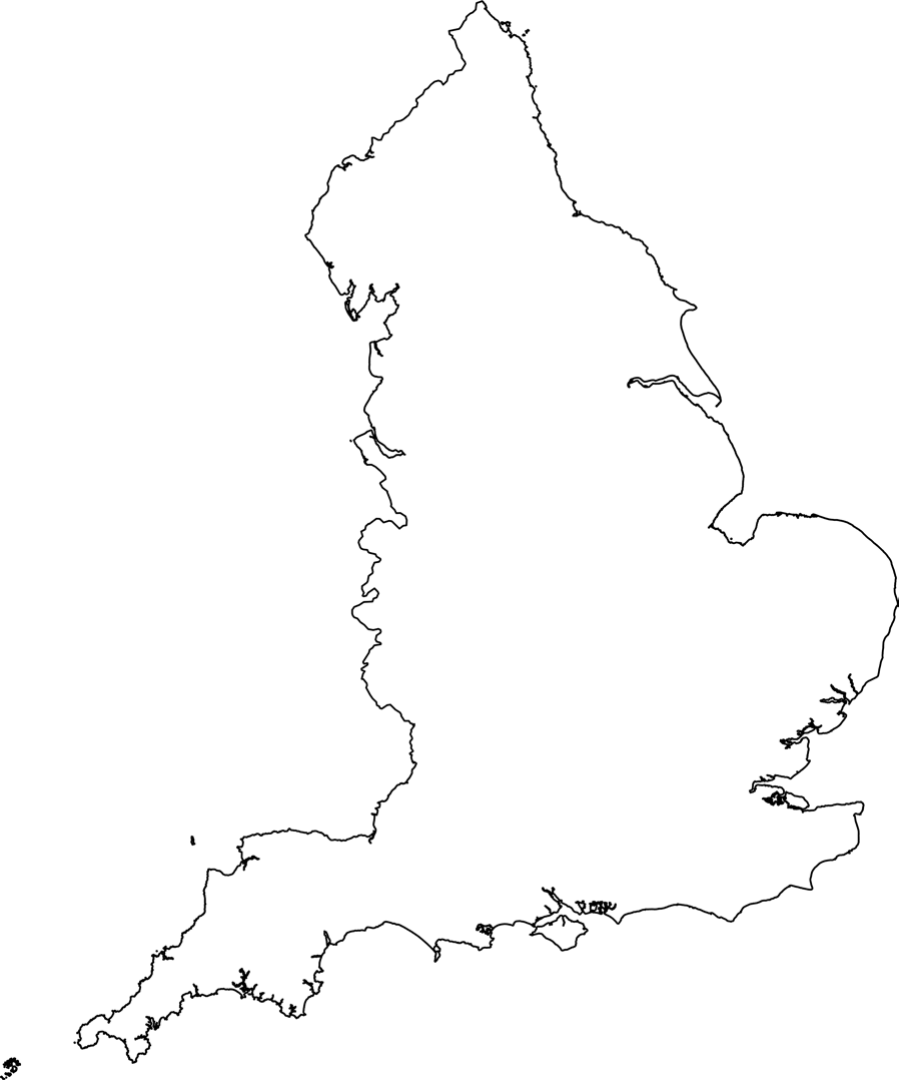
Geographical boundaries of England

First, the package maptools [23] for reading and manipulating geographic data is loaded. After that, the commonly used coordinate reference system WGS84 is chosen. The shapefile of the United Kingdom is imported, and finally, England is selected.^2^ Figure 3 shows the administrative boundaries of England.

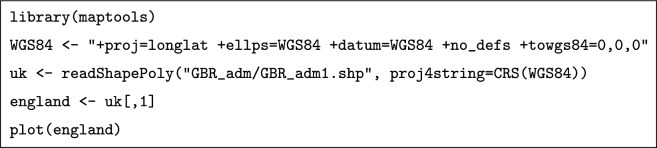



#### Step 2: Preprocessing and geocoding of residential and hospital addresses

The files containing address information on residents and hospitals are loaded. The package ggmap [25] was used to query the longitude and latitude of these address information from Google. The administrative database contains addresses of the United Kingdom. Therefore, Scotland, Belfast (covers all of Northern Ireland), Isle of Man, Guernsey, and Jersey were removed based on the postal code area. Removing these areas resulted in approximately 12 million remaining addresses. From those, a random sample of 13,000 addresses was drawn from this database, and their geo-coordinates were queried.

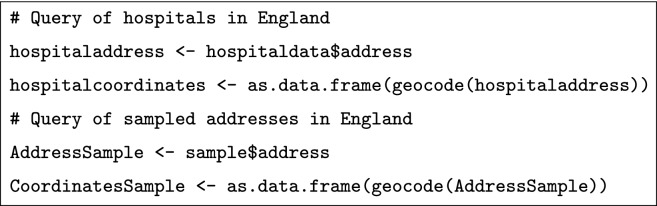



**Fig. 4:**
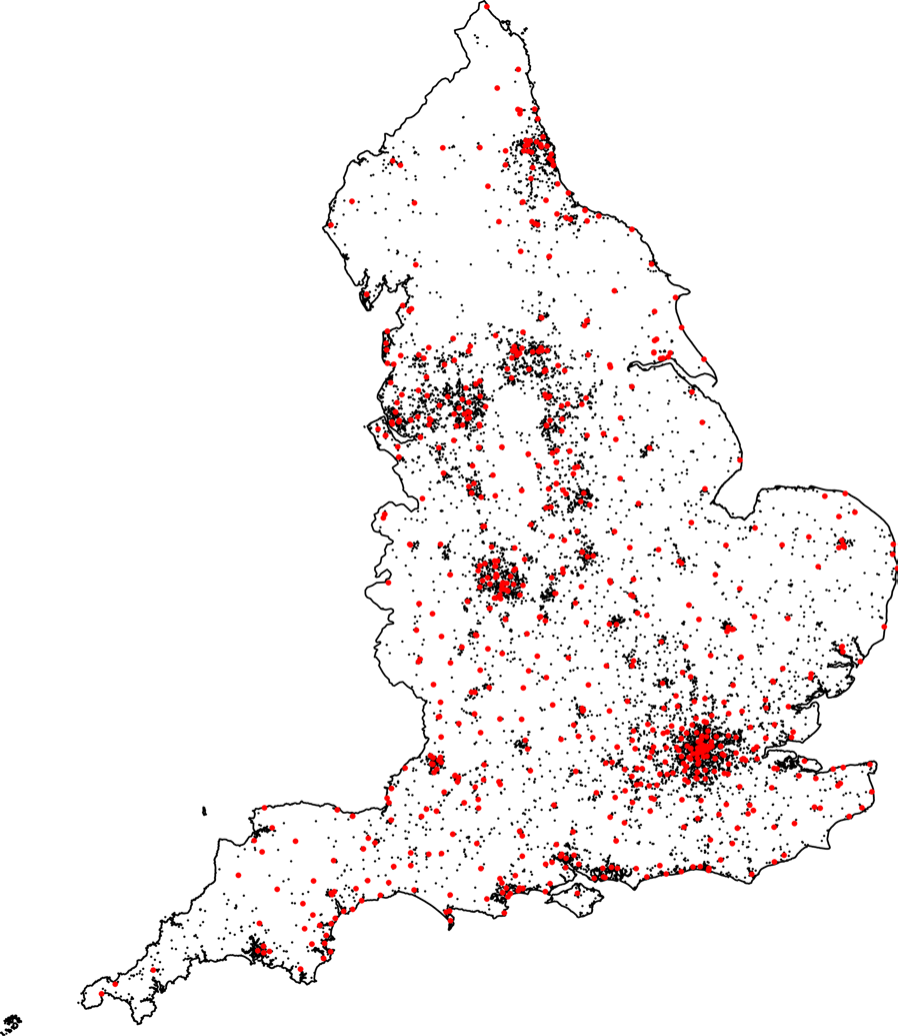
Queried hospital (in red) and residential addresses (in black)

Some of the sampled addresses resulted in incorrect queries due to the administrative database being deprecated. Those addresses were removed from the analysis. Further, it was verified whether all successful queries are within the administrative boundaries of England. Therefore, the function over from the package sp [26] was used. Removing these coordinates reduced the number of residential addresses considered to 12,057. The final result of preprocessing hospital and residential data is shown in Fig.  4.

#### Step 3: Enlarge area considered for computation

**Fig. 5:**
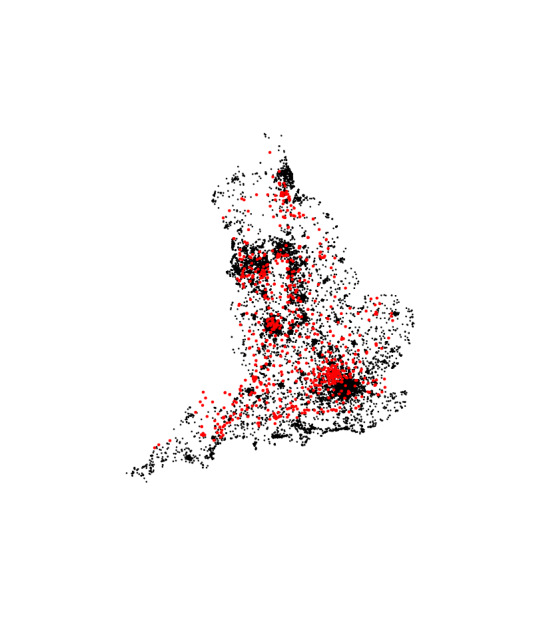
Enlarged geographical area with addresses of hospitals (in red) and residential addresses (in black)

The boundaries of England are enlarged for computation. For coordinates close to the geographical boundaries of England, the circles drawn will include fewer grid points. This will cause a loss in precision of the approximation. Further, in such scenarios, the risk for re-identification might increase. The surface of England is approximately $130,300\,\text {km}^2$. The surface of the enlarged area is $1,490,000\,\text {km}^2$. The artificially generated area covers England 11 times and preserves the underlying geographical structure of the addresses and England, respectively. Figure 5 shows the generated expanded geographical area with hospitals and addresses. This step is optional.

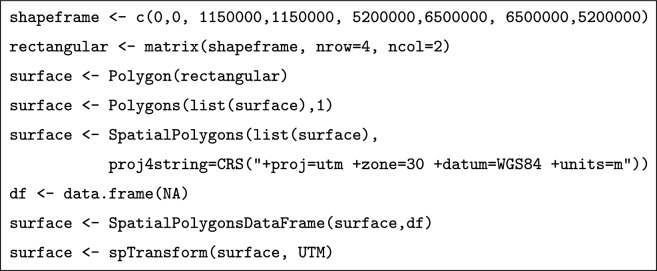



#### Step 4: Change of coordinate system

Although calculation of geographic distances from the WGS84 coordinates is possible with the R package sp [26], an approach using Euclidean distances is sufficient here, since the considered area is small. Therefore, the WGS84 coordinates are transformed to UTM coordinates using the package rgdal [27].

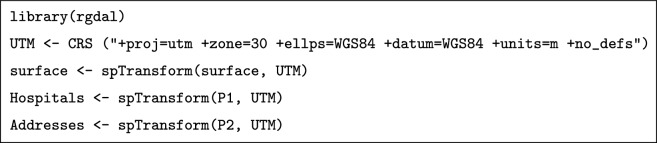



#### Step 5: Selection of coordinates

For demonstration purposes and the further steps in the example, we restrict the data shown in Fig. 5 to Cornwall and Devon (South West England) and four arbitrary chosen coordinates. One residential address and its nearest three hospitals. This step is for demonstration purposes only and not necessary for the method to work.

#### Step 6: Grid generation

**Fig. 6:**
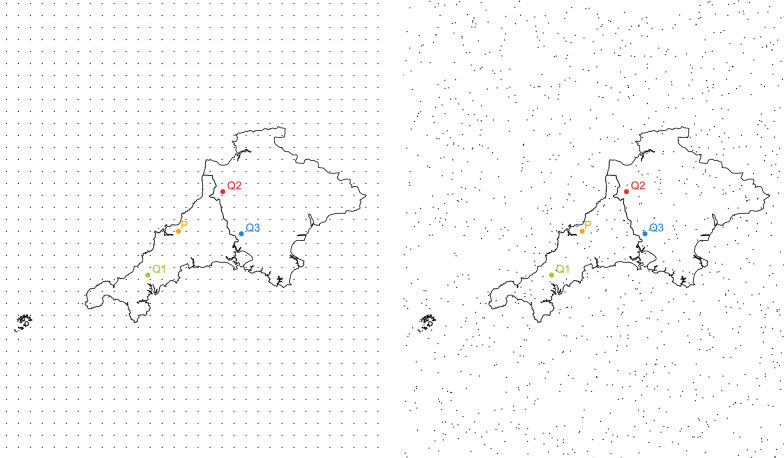
Regular (left panel) and random grid (right panel) generation. Residential address *P* in orange and three nearest hospitals *Q*1, *Q*2, *Q*3 in green, red, and blue

As mentioned before, either randomly or regularly distributed grid points may be used. The R package sp contains functions for the generation of both regular and random grids. As an example grids consisting of $n = 20,000$ grid points, randomly sampled from the enlarged geographical area, were generated. In Fig. 6, a grid with regularly distributed grid points is compared with a grid consisting of randomly distributed grid points.

#### Step 7: Assignment of random numbers to the grid points

**Fig. 7:**
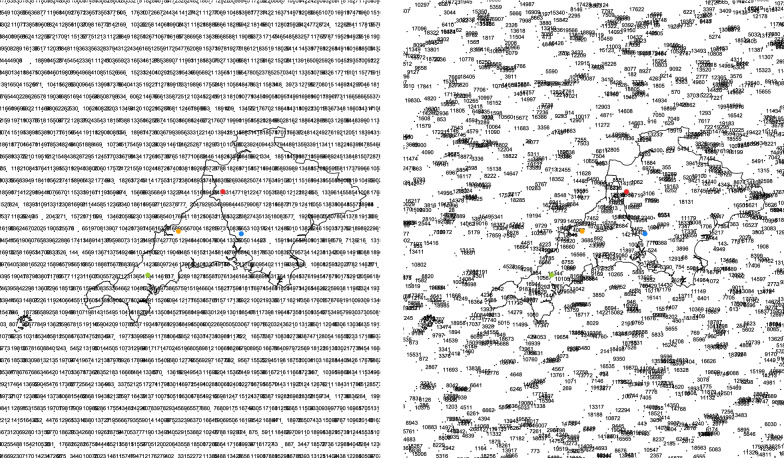
Regular (left panel) and random grid (right panel) generation with assignment of random numbers. Residential address in orange and three nearest hospitals in green, red, and blue

The next step consists in randomly assigning the arbitrarily chosen numbers 1, ..., 20,000 to the grid points. See Fig.  7 for the result (only the part covering Cornwall and Devon is shown).

#### Step 8: Determination of pseudonymizations

**Fig. 8:**
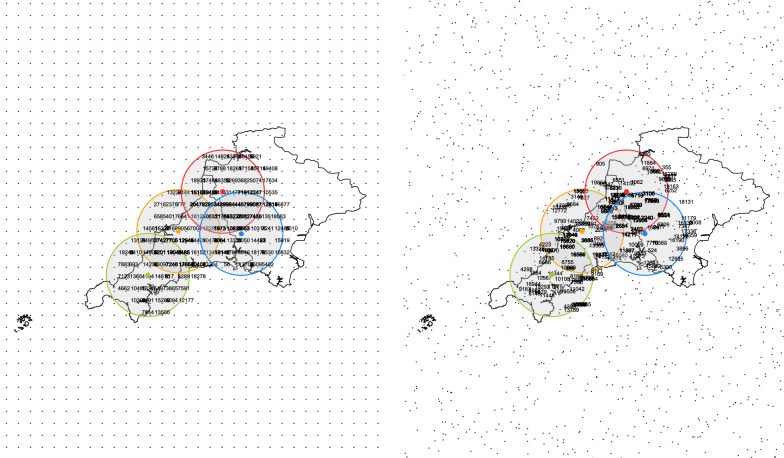
Regular (left panel) and random grid (right panel) with circles around example addresses with labeled random numbers. Residential address in orange and three nearest hospitals in green, red, and blue

The R package sp provides functions for calculating spatial distances between points. At first, the distances between *P* and each grid point, as well as the distances between *Q* and each grid point are calculated. Next, for each of the points, *P* and *Q*, a set of integers is determined. This set depends on a parameter *r*, which denotes the radius of a circle (in meters) with center *P* and *Q*, respectively (see Fig. 8). The resulting set consists of the random labels of those grid points, which have a distance less than *r* from the respective point *P*, *Q*. Here, the radius (*r*) is set to 30 km. The following R code shows the pseudonymization of *P* and $Q_1$.

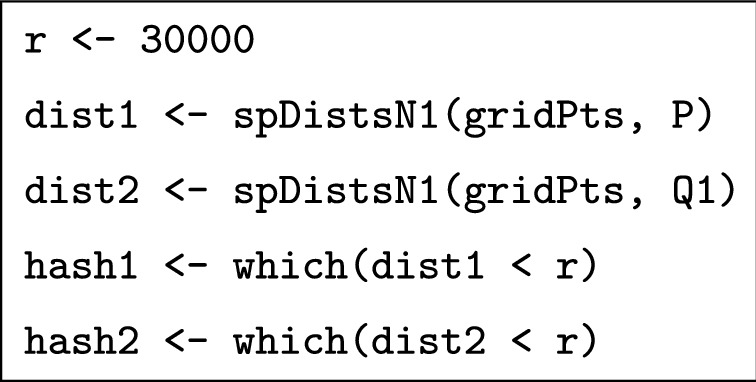



#### Step 9: Computations of the approximate distance

This is the only step necessary for a research group interested in the distances. The Dice coefficient [cf. Sect. 2, Eq. (2)] of the two sets of grid points enclosed by the two circles can be computed directly:

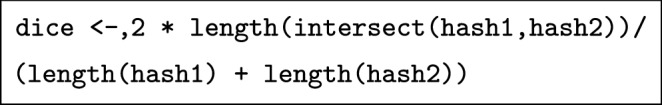



The Dice coefficients for *P* and $Q_1, Q_2, Q_3$ are shown in Table 1.

**Table 1:** Dice coefficients by grid type

Geo-locations	Dice regular grid	Dice random grid
$\{P, Q1\}$	0.234	0.154
$\{P, Q2\}$	0.179	0.217
$\{P, Q3\}$	0.132	0.112

The remaining problem is the computation of the approximated distance given the already computed similarity of the two sets of grid points. Regardless which kind of grid is being used, the area of intersection *A* between the two circles with radius *r* around the considered points can be estimated by the command.

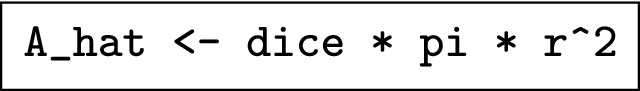



[cf. Sect. 2, Eq. (3)]. As described in Sect. 2, Eq. (1), the area of intersection depends on *d* and we can approximate the distance between *P* and *Q* by solving Eq. (1). The R package stats provides the function uniroot, which searches the interval from lower to upper (the closed interval [0, 2*r*]) for a root (i.e., zero) of the considered function ($A(d)-\hat{A}$) with respect to its first argument (*d*) and with accuracy tol ($1 \cdot 10^{-9}$). To use the function uniroot the function to estimate the area of the intersection has to be defined with the command

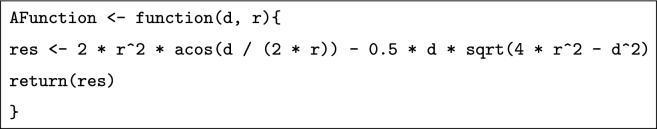



Finally, the desired approximations of the distances can be computed with the following command:

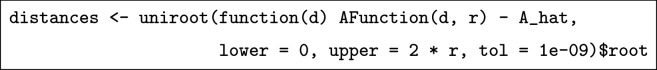



Please note, that only A_hat (estimated area of intersection) and the parameter *r* are needed as input.

For the example given, the original distances, the approximated distances, and relative errors for both, regular and random grids, are shown in Table 2.

**Table 2:** Results of distance approximations

Set of	Original *d* (m)	Approximated *d* (m)	Relative error	Approximated *d* (m)	Relative error
addresses		regular grid	regular grid	random grid	random grid
$\{P, Q_1\}$	38,539	39,081	$-0.014$	44,326	$-0.131$
$\{P, Q_2\}$	42,883	42,573	0.007	40,108	0.069
$\{P, Q_3\}$	45,367	45,918	$-0.012$	47,358	$-0.042$

Thus, in the example given, the absolute relative error is about 1% for the approximations using the regular grid and varies between 4% and 13% for the random grid. However, these are just a few numerical examples. In general, the size of the errors depends on the radius and the number of grid points used. For a fixed radius, the number of common grid points of the circles around *P* and *Q* strongly depends on the number of grid points sampled from the area of intersection. In contrast, there is nearly the same number of grid points enclosed by the area of intersection between the two circles in each run for the regular grids (the two plots illustrate this in Fig. 8). Accordingly, more accurate results can be expected using regular grids. Therefore, only the regular grid is considered in the following simulation. Moreover, the mean error of random grids will approach the mean error of regular grids with increasing radius since more grid points will be in the intersect.

## Results

We systematically studied the effect of different choices of numbers of grid points and radii on the quality of the approximations in a full factorial simulation experiment (number of grid points, radius). Therefore, the data described in Sect. 2 is used. For each residential address, the distances to its nearest three hospitals were approximated. As parameters, radii between 10–100 km by steps of 10 km and number of grid points between 50,000–100,000 by steps of 10,000 were used. A large number of grid points is required due to enlarging the original geographic area to avoid empty intersects.

**Fig. 9:**
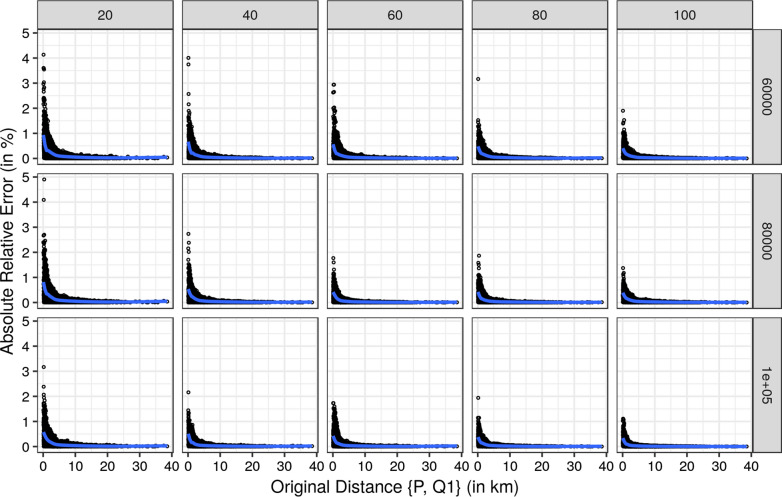
Relative error of the approximated distances for the nearest hospital by size of radius and number of grid points. The blue line is a smoother using gam [28]

**Fig. 10:**
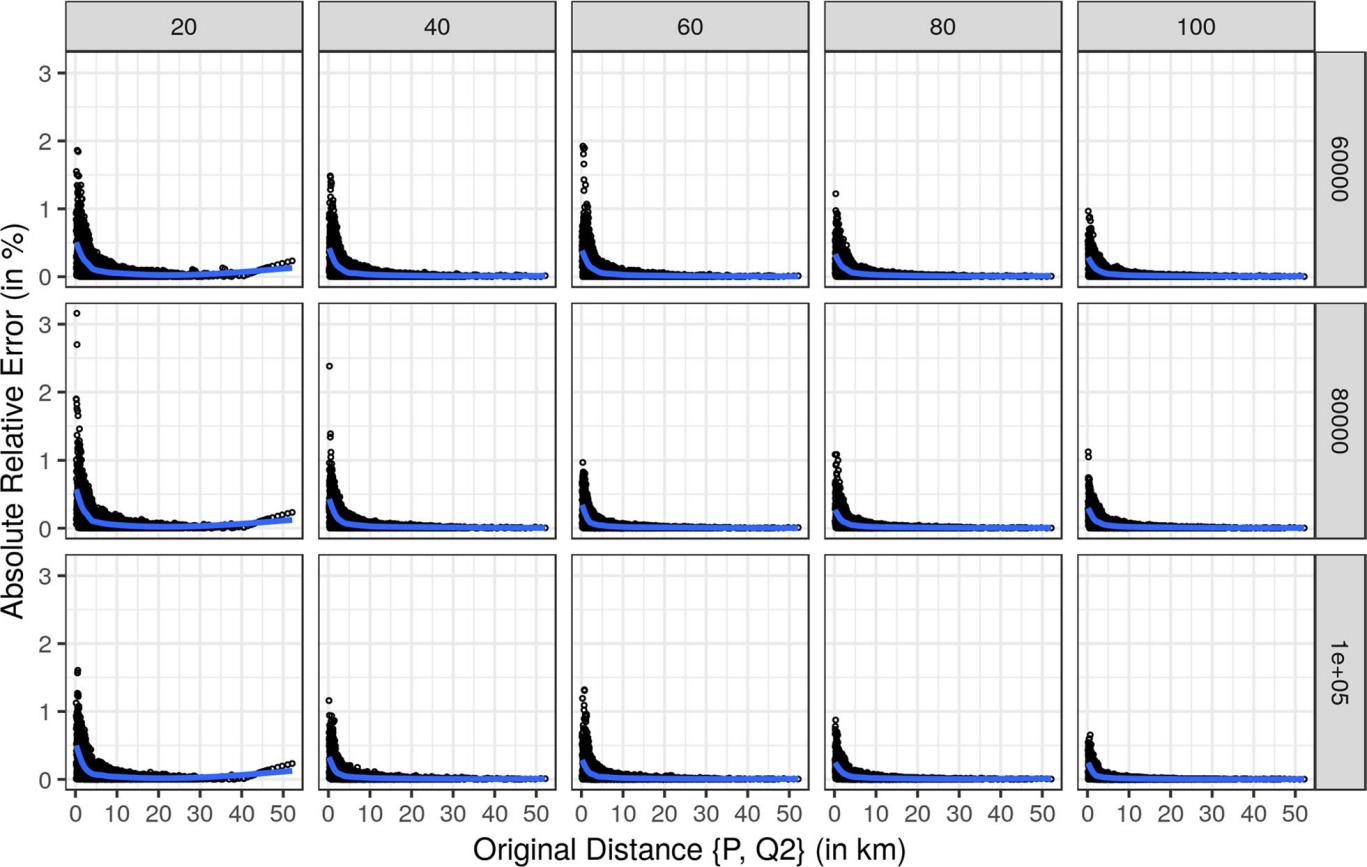
Relative error of the approximated distances for the second nearest hospital by size of radius and number of grid points. The blue line is a smoother using gam

**Fig. 11:**
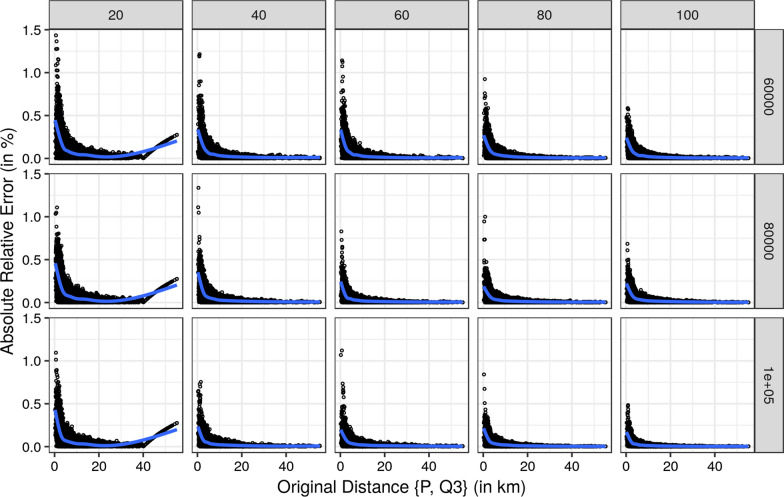
Relative error of the approximated distances for the third nearest hospital by size of radius and number of grid points. The blue line is a smoother using gam

First, we report the results comparing the original distances and the corresponding absolute relative error for each approximation individually for each of the three nearest hospitals. For a more concise presentation we restricted Figs. 9, 10 and 11 to radii of 20, 40, 60, 80, and 100 km and to 60,000, 80,000, and and 100,000 grid points.

All three Figures show the same pattern. Smaller original distances have larger absolute relative errors, which decrease with increasing original distances. The largest absolute relative errors resulted for the nearest hospital with smaller radii and fewer grid points. However, the absolute relative error does not exceed 5% for the nearest hospital. Hence, the absolute relative errors for the second and third nearest hospitals are below 5%. With an increasing number of grid points and increasing radii size, the quality of the approximations increases since the absolute relative errors are decreasing.

Despite the small error in the approximations, about 11% of the orders of precedence in the hospitals were not preserved. This is mainly due to the small differences in distances between nearest and second nearest hospital.

**Fig. 12:**
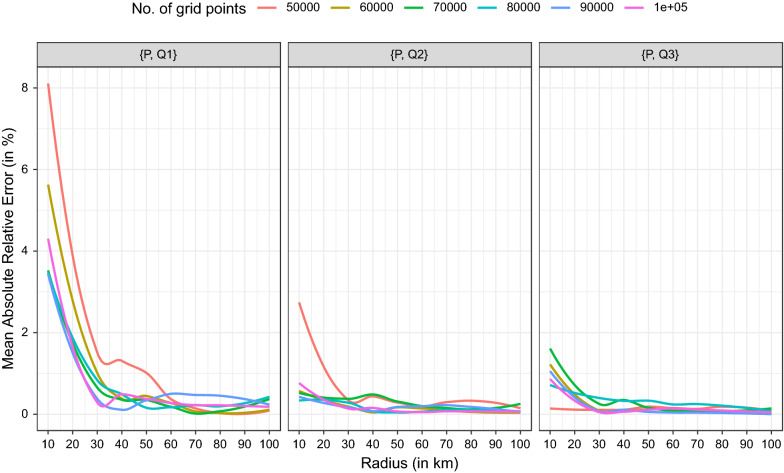
Mean absolute relative error for each of the three nearest hospitals

Second, we report aggregated results based on the entire parameter space (see Fig. 12). Here, the mean absolute error by radii and the number of grid points for each of the three nearest hospitals are shown.

The largest mean absolute relative error of about 8% is observed for 50,000 grid points and a radius of 10 km. The effect of the number of grid points on the quality of the approximations decreases with the size of the radius. Further, the effect of the number of grid points on the quality of the approximations also vanishes with larger distances. With a radius size of about 30 km or more, no major differences in the errors remain. With the data used, mean absolute relative errors $<1\%$ can be achieved using a radius of $\ge 30$km and $\ge 60,000$ grid points. Table 3 shows the errors (in meters) for two parameter sets. For the suboptimal parameter set, the errors (in meter) are already small. With an optimal parameter set, minor errors of about 100m can be achieved and are negligible in practical applications.

**Table 3:** Results of distance approximations by radius and number of grid points

Geo-locations	Radius in (m)	Grid points	Mean absolute error in (m)
$\{P, Q1\}$	10,000	50,000	791
$\{P, Q2\}$	10,000	50,000	1044
$\{P, Q3\}$	10,000	50,000	1422
$\{P, Q1\}$	90,000	100,000	136
$\{P, Q2\}$	90,000	100,000	140
$\{P, Q3\}$	90,000	100,000	2141

Hence, higher numbers of grid points on a regular grid will yield small errors. The choice of the radius is crucial for small numbers of grid points. Furthermore, it should be noted that the variances of errors of approximated distances for fixed radii and fixed numbers of grid points are very small.

Of course, the choice of radii is critical: for unsuitable radii, the mean error gets unsustainable high. However, the radius (*r*) is a user-defined parameter. For many practical applications, distances above a certain threshold are considered as irrelevant. Often points in the upper tail of the distribution of distances can be censored (for example: all distances over 100 km), and this could be considered the maximum distance of interest. In general, the radius should be at least half the maximum distance of interest. ISGP allows distance calculations for points separated by a distance less than 2*r*. For points separated by more than 2*r*, only the fact that the distance is ’2*r* or greater’ can be stated. Since *r* is user-defined, this is not an issue.

**Fig. 13:**
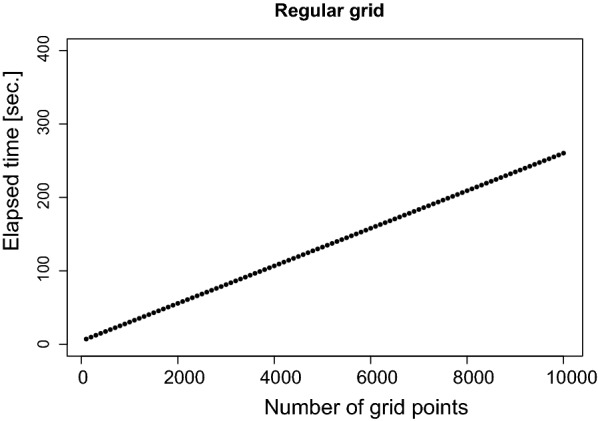
Elapsed time in seconds depending on the number of grid points

The runtime needed for the computation of the distance approximation is a linear function of the number of grid points (see Fig.  13). The runtime is unrelated to the radii. Overall, currently about 10,000 approximations can be computed within less than 5  min for regular grids. An advantage of the method is that even with large numbers of grid points, storage is no limitation because 10,000 points can be stored in less than 20 kB.

## Discussion and conclusion

In this paper, we have introduced ISGP as a method for the calculation of the distance between masked geographical data. ISGP guarantees high security since an adversary could only uncover sets of random numbers, but not the original locations.

In principle, all geo-masking methods can be attacked with a graph-theoretical approach, if a distance matrix and restricting additional information is available [18, 29]. If the elements of the distance matrix are censored, such approaches become more difficult. Since distances above 2*r* will result in empty intersections, only distances smaller than 2*r* can be computed. Therefore, given a dataset with *n* observations, only distances smaller than 2*r* of a $n *n$ distance matrix can be recovered. Hence, graph-theoretical attacks on distance matrices of randomly labeled grid points should be much more difficult than on uncensored distance matrices. However, a detailed security analysis of ISGP will be the topic of future research.

We have demonstrated that the method provides acceptable results. For the intended applications, relative errors between a minimum of approximately 1 % and a maximum of 10 % are acceptable. The effect of approximately 10 % random measurement error on correlations is negligible for most practical applications. If we are interested in the correlation between true distances and a criterion variable (for example medical outcomes), but we observe only approximated distances, the reliability of the true (*x*) and approximated (${\widehat{x}}$) distances will be $\rho (x,{\widehat{x}})=\frac{\sigma _{x}^{2}}{\sigma _{\hat{x}}^{2}}$. Using this reliability value with an expected error of 10 %, even after correction for attenuation, the decrease in correlations is negligible for most practical applications. The amount of attenuation due to the approximation will be smaller than that. However, in the area of non-emergency medical care, variations in the travel of less than thirty minutes in general do not cause serious complications [30].

A further advantage of the described approach is the prospect to use IGSP encoded geographical information for privacy preserving record-linkage (PPRL) applications (for a review, see [31]). Similar to the ordinality preserving mapping of numerical values described by [13], the resulting set of grid numbers of IGSP could be mapped to Bloom-filters [32]. Bloom-filters are increasingly used in PPRL [11, 33, 34] and could be enhanced with ordinal encoded geographical data by ISGP [14]. Bloom-filter encoded IGSP are currently the only PPRL method, which can efficiently utilize geographical information. A detailed study on this application will be the topic of a forthcoming paper.

To sum up, we discussed a new method for privacy protection of geographical information in microdata. The use of intersecting sets of randomly labeled points permits fast distance approximations with errors below 10 %, where larger errors are due to unsuitable parameter choices. With appropriately chosen radii, about 99 % accuracy can be achieved. However, a systematic comparative study of the accuracy and privacy of geomasking methods, in general, is lacking in the literature and subject of ongoing research. Furthermore, the technique as described here is limited to Euclidean distances. To account for differences between actual driving time and driving time according to the Euclidean distance, we are working on mapping these differences by using more than two dimensions of the random grid. This technique will be the subject of a forthcoming paper.

ISGP neither requires unduly computational effort nor excessive storage. The method will be useful for research using geo-located sensitive data.^3^

## Figures and Tables

**Fig. 14:**
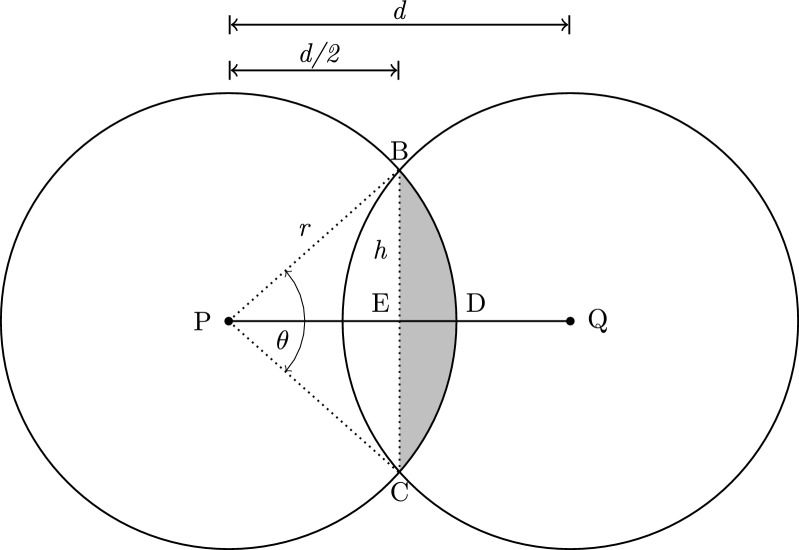
Area of intersection between two circles with the same radius *r*, whose centres *P* and *Q* have distance *d*

## Data Availability

The datasets supporting the conclusions of this article are available at [22, 24] or can be made available from the corresponding author on request. The data containing residential geographic information cannot be made publicly available to protect privacy and consent. The code that supports the conclusions of this study is available in the paper.

## Appendix: equation for the intersection of two circles

In this appendix we provide a geometrical proof of the formula of the area of intersection of two similar circles. As mentioned, the area depends on the distance *d* between the centres *P* and *Q*, cf. Sect. 3, Eq.  (1).

Let $r \in {\mathbb {R}}$ be the respective radius of two circles and $d \in [0,2r]$ the distance between their centers. The area of intersection of the two circles can be calculated by

$$\begin{aligned} A(d)= 2r^2 \cdot \cos ^{-1} \left( \frac{d}{2r} \right) -\frac{d}{2} \cdot \sqrt{4r^2-d^2}. \end{aligned}$$

### Proof

The area of the shaded segment *BDC* is the area of the sector *PBDC* minus the area of the triangle $\triangle$ *PBEC* (see Fig. 14). To determine these areas, we need the enclosed angle $\theta$, which is by definition:

$$\begin{aligned} \cos \frac{\theta }{2}=\frac{PE}{PB}=\frac{d}{2r}. \end{aligned}$$

Hence

4 $$\begin{aligned} \frac{\theta }{2}=\cos ^{-1} \frac{d}{2r}. \end{aligned}$$

For the area $\triangle PBEC$ we need the length *BC*. Since $BC/2=EB$, we can use the triangle $\triangle$ *PBE* and the theorem of Pythagoras to get

5 $$\begin{aligned} h=EB=\sqrt{r^2-\frac{d^2}{4}} \end{aligned}$$

and finally

$$\begin{aligned} area(\triangle PBEC) = \frac{1}{2} \left( 2h \cdot \frac{d}{2} \right) = \frac{hd}{2}. \end{aligned}$$

Using radiants, a full circle has an angle of $2\pi$ and an area of $\pi \cdot r^2$. Since a sector is a slice with an angle of $\theta$, the sector has an area proportional to the angle $\theta$:

This gives

Inserting Eq. (4) for $\theta$ and eq. (5) for *h* gives

The area of overlap *A*(*d*) is twice the area of the segment:

$$\square$$

## Footnotes

1 The file is publicly available and was downloaded from [22].

2 The shapefile containing the administrative boundaries of the United Kingdom was downloaded from [24]. The website provides administrative boundaries of many more countries, so that the workflow can be easily adapted to other countries or continents.

3 We used the proposed method in a first real-world application in a study of regional disparities of educational opportunities [12]. However, that publication does not contain any technical details which we provided in this paper.
